# Baseline Characteristics of Study Participants in the Early Life Interventions for Childhood Growth and Development in Tanzania (ELICIT) Trial

**DOI:** 10.4269/ajtmh.19-0918

**Published:** 2020-08-10

**Authors:** Tarina C. Parpia, Sarah E. Elwood, Rebecca J. Scharf, Joann M. McDermid, Anne W. Wanjuhi, Elizabeth T. Rogawski McQuade, Jean Gratz, Erling Svensen, Jonathan R. Swann, Jeffrey R. Donowitz, Samwel Jatosh, Siphael Katengu, Paschal Mdoe, Sokoine Kivuyo, Eric R. Houpt, Mark D. DeBoer, Estomih Mduma, James A. Platts-Mills

**Affiliations:** 1 Division of Infectious Diseases and International Health, University of Virginia, Charlottesville, Virginia;; 2 Department of Pediatrics, University of Virginia, Charlottesville, Virginia;; 3 Haukeland University Hospital, Bergen, Norway;; 4 School of Human Development and Health, Faculty of Medicine, University of Southampton, Southampton, United Kingdom;; 5 Division of Infectious Disease, Children’s Hospital of Richmond at Virginia Commonwealth University, Richmond, Virginia;; 6 Haydom Global Health Research Centre, Haydom Lutheran Hospital, Haydom, Tanzania;; 7 National Institute for Medical Research, Muhimbili Medical Research Centre, Dar es Salaam, Tanzania

## Abstract

Recurrent enteric infections and micronutrient deficiencies, including deficiencies in the tryptophan–kynurenine–niacin pathway, have been associated with environmental enteric dysfunction, potentially contributing to poor child growth and development. We are conducting a randomized, placebo-controlled, 2 × 2 factorial interventional trial in a rural population in Haydom, Tanzania, to determine the effect of 1) antimicrobials (azithromycin and nitazoxanide) and/or 2) nicotinamide, a niacin vitamer, on attained length at 18 months. Mother/infant dyads were enrolled within 14 days of the infant’s birth from September 2017 to September 2018, with the follow-up to be completed in February 2020. Here, we describe the baseline characteristics of the study cohort, risk factors for low enrollment weight, and neonatal adverse events (AEs). Risk factors for a low enrollment weight included being a firstborn child (−0.54 difference in weight-for-age *z*-score [WAZ] versus other children, 95% CI: −0.71, −0.37), lower socioeconomic status (−0.28, 95% CI: −0.43, −0.12 difference in WAZ), and birth during the preharvest season (November to March) (−0.22, 95% CI: −0.33, −0.11 difference in WAZ). The most common neonatal serious AEs were respiratory tract infections and neonatal sepsis (2.2 and 1.4 events per 100 child-months, respectively). The study cohort represents a high-risk population for whom interventions to improve child growth and development are urgently needed. Further analyses are needed to understand the persistent impacts of seasonal malnutrition and the interactions between seasonality, socioeconomic status, and the study interventions.

## INTRODUCTION

Poor child growth is an intractable problem in resource-poor settings and has been associated with poor cognitive development, decreased school performance, and lower economic productivity.^1–3^ Haydom, Tanzania, a rural, agricultural town, was one of eight sites included in the Etiology, Risk Factors, and Interactions of Enteric Infection and Malnutrition and the Consequences for Child Health (MAL-ED) study evaluating correlates of childhood growth and development from 2009 to 2014.^4^ The prevalence of stunting in the Haydom MAL-ED cohort was found to be 70% at 18 months of age.^5,6^ Although water, sanitation, and hygiene (WASH) interventions and micro- or macronutrient supplementation are widely thought to contribute to stunting, the efficacy of interventions to improve these factors has been inconsistent or limited.^7–11^ There is evidence that recurrent enteric infections and micronutrient deficiencies, including deficiencies in the tryptophan–kynurenine–niacin pathway, are associated with environmental enteric dysfunction, which may contribute to poor child growth and stunting. Clinically, a deficiency of niacin and the essential amino acid tryptophan may manifest as pellagra.^2,5,12–15^ We have previously described the rationale and design of the Early Life Interventions for Childhood Growth and Development in Tanzania (ELICIT) study, an ongoing interventional trial to determine the effect of antimicrobials and micronutrient supplementation on linear growth in children in Haydom.^6^

Because the study population in Haydom and the surrounding area live in a region with a unimodal crop cycle and a maize predominant diet, they may be particularly predisposed to nutritional deficiencies, including niacin. Seasonal patterns of food insecurity and hunger have been shown to significantly impact birth weight and rates of acute malnutrition in this area.^16^ Birth season, typically defined in relation to the harvest, has been associated with long-term negative consequences on growth and educational attainment in Tanzania and is more predictive of these outcomes than average food consumption.^17^ During the preharvest period, a time when the previous year’s harvest stocks are diminished, both diet diversity and the quantity of food consumed are reduced.^18^ This preharvest season is also associated with seasonal peaks in *Shigella* infections, which have been associated with poor growth.^15,19^ It is, therefore, possible that the study interventions may have a greater impact in the preharvest season. Because the impact of interventions to improve child growth and development may be poorly generalizable across populations, we describe the baseline characteristics of the ELICIT study population here in detail, compare these with the MAL-ED cohort, and describe seasonal and other risk factors for low enrollment weight, used as a baseline marker of prenatal risk for poor long-term growth outcomes.

## MATERIALS AND METHODS

The ELICIT design and protocol is described in detail elsewhere.^6^ In brief, the study is a randomized, placebo-controlled, interventional trial to determine whether administration of 1) antimicrobials (azithromycin and nitazoxanide) to reduce the subclinical carriage of enteric pathogens and/or 2) nicotinamide, a niacin vitamer, to reduce intestinal inflammation^14^ has an effect on linear growth at 18 months. Mothers could provide informed consent during pregnancy, but mother/child dyads were enrolled within 14 days of the child’s birth from September 2017 to September 2018, when the inclusion and exclusion criteria were met. Specifically, participants were eligible for enrollment if the mother was 18 years of age or older and if the child was 14 days of age or younger and born in the Haydom catchment area. Participants were excluded for maternal inability to adhere to the protocol (i.e., is not able to follow directions from the field team), multiple gestation, significant birth defect or neonatal illness, weight < 1500 g at enrollment (age ≤ 14 days), lack of intent to breastfeed the infant, and plan to move from the area within the subsequent 18 months.^6^ As a 2 × 2 factorial study design, dyads were randomized to one of four arms: placebo + placebo, azithromycin and nitazoxanide + placebo, placebo + nicotinamide, or azithromycin and nitazoxanide + nicotinamide. Mothers were given daily nicotinamide (or placebo) from birth through 6 months. Children received daily nicotinamide (or placebo) starting at 6 months. Azithromycin and nitazoxanide (or placebo) were given at 6, 9, 12, and 15 months, or 12 and 15 months, respectively. In addition to receiving interventions, standardized questionnaires were administered at each monthly visit to document maternal reports of illness, healthcare-seeking behavior, infant feeding practices, and dietary information including food insecurity. A broad sociodemographic questionnaire was administered at the first monthly visit, including questions about the household composition, WASH, income, family assets, and exposure to animals. All enrolled children who completed this first visit, regardless of subsequent follow-up status, were included in this analysis.

Household characteristics were previously compiled for children enrolled in the MAL-ED study (2009–2014) from a similar geographic area. Characteristics of the households in the greater Manyara Region, in which most of the ELICIT and MAL-ED study populations reside, were compiled from the 2015–2016 Tanzania Demographic and Health Survey and Malaria Indicator Survey (DHS), a nationally representative household survey.^20^

### Study setting.

**Figure 1.**
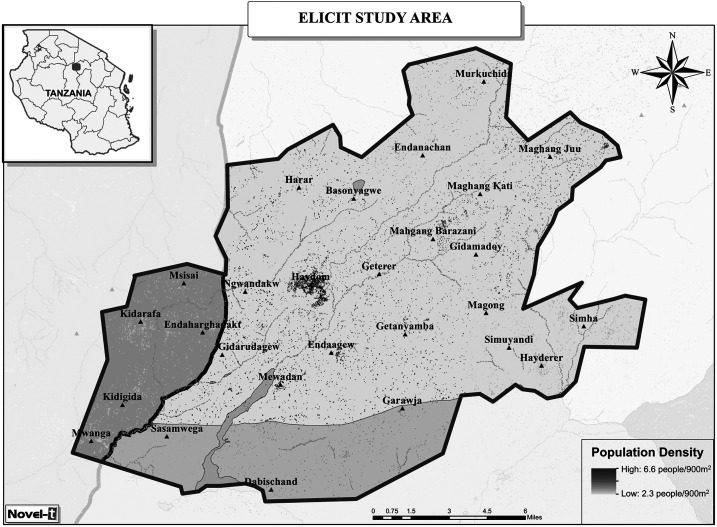
Early Life Interventions for Childhood Growth and Development in Tanzania study area. Shading and outlines indicate the three districts and two regions included in the study area (lightest shading: Mbulu/Manyara; intermediate shading: Hananga/Manyara; darkest shading: Mkalama/Singida).

Haydom is located in the Manyara (north-central) region of Tanzania. Haydom Lutheran Hospital, Haydom town, is the largest hospital in the Mbulu district. The study population lives primarily in the Manyara Region as well as in a small portion of the Singida region, within an approximately 25-km radius from Haydom town (Figure 1). The area is ethnically and geographically diverse and relies primarily on subsistence agriculture. There is one annual harvest starting in approximately April of each year.^4,15^ Of note, the MAL-ED catchment area did not include Haydom town.

### Variable definitions.

Improved sanitation and drinking water sources were defined based on the WHO guidelines.^21^ An improved drinking water source included a household connection, public standpipe, borehole, protected dug well, protected spring, or rainwater collection. Improved sanitation facilities were connected to a public sewer or septic system, a pour-flush latrine, or a ventilated improved pit latrine. Treated water referred to water that was boiled, filtered, or treated with bleach. The Water and sanitation, Assets, Maternal education, and household Income (WAMI) score, calculated for children in both MAL-ED and ELICIT, is a composite measure of socioeconomic status derived using data from all eight MAL-ED sites.^22^ Crowding was defined as two or more people sleeping per room in the household.

Anthropometry was performed as previously described.^6^ In brief, all field team members were trained in the accurate measurement of anthropometrics and a standardized operating protocol was used for all measurement procedures. The enrollment weight, in kilograms, was measured to the nearest 10 g using a digital scale. Length was measured to the nearest millimeter by two research field-workers with the child lying flat on a measuring board with a fixed perpendicular board against which the head was placed and a second adjustable perpendicular board was placed against the feet. An average of two measurements was used, if within 2 mm of each other. If not, a third measurement was obtained, and the average of the two closest measurements was used. Head circumference, in centimeters, was measured to the nearest millimeter by placing a non-distensible tape around the child’s head, above the eyebrows, above the ears, and around the biggest part of the back of the head. Weight-for-age *z*-score (WAZ), length-for-age *z*-score (LAZ), and head circumference-for-age *z*-score (HCZ) were calculated per the 2006 WHO growth standards,^23^ using the zscorer package version 0.2.0 in *R*.^24^ *Z* score values were then cleaned by first identifying outliers defined as values outside of the 0.5th percentile, which were then removed if not consistent with subsequent measurements.

Exclusive breastfeeding status in the ELICIT trial, asked at the first monthly visit, was defined as current breastfeeding without any introduction of liquids or solid food into the diet in the previous month. To assess for food insecurity, mothers were asked, “Since the last visit, has the mother worried that the household would not have enough food?” Mothers could respond “No,” “Rarely,” “Sometimes,” or “Often.” We converted this into a binary indicator to compare those who had no concern over food availability with those who expressed any concern. Using data available from both the MAL-ED and ELICIT cohorts, the preharvest season was defined as the period of consistently high food insecurity, from November to March, ending with the harvest in April.

Adverse events (AEs) and serious adverse events (SAEs) that occurred in the first month of life were also recorded. An AE was defined as any untoward or unfavorable medical occurrence in a participant, including any abnormal sign (e.g., abnormal physical examination or laboratory finding), symptom (e.g., maternal report during a monthly study visit), or disease even if the event is not considered to be related to the investigational interventions. An SAE was defined as an event that resulted in any of the following outcomes: death, life-threatening illness, hospitalization or prolongation of hospitalization, an event that resulted in persistent or significant incapacity/disability, an event that was medically significant and which the investigator regarded as serious based on medical judgment, or a laboratory result on the safety laboratory test that was in the range for SAE. All SAE reports were reviewed by a pediatrician to ensure appropriate event categorization.

### Statistical analysis.

Baseline characteristics were reported using frequencies and means. Summary statistics for major socioeconomic and demographic indicators were tabulated for ELICIT, MAL-ED, and the DHS where questions were comparable enough to be reasonably certain the data were capturing the same classification. Univariable and multivariable linear regression models were performed to assess factors influencing enrollment WAZ and LAZ. However, pre- and postharvest seasons were used rather than individual calendar month to the multivariable model. Poisson approximation to log-binomial regression with robust standard errors was used to estimate risk of AEs and SAEs. Statistical analyses were performed using *R* version 3.5.2.

## RESULTS

In total, 1,205 children were assessed for eligibility, of whom 1,188 (98.6%) were enrolled and 1,170 (97.1%) completed the first monthly visit. In comparison to a previous cohort study conducted in the same region (MAL-ED), there was evidence of socioeconomic improvements in the population (Table 1). In ELICIT, a higher proportion of mothers had greater than or equal to 7 years of education (15.5% increase, 95% CI: 9.1%, 22.1%) and reported exclusive breastfeeding in the first month (9.2% increase, 95% CI: 5.1%, 14.3%). In addition, more households reported an improved drinking water source in ELICIT (25.7% increase, 95% CI: 19.0%, 32.2%), although a decreased proportion reported access to an improved latrine (5.2% decrease, 95% CI: 0.7%, 10.5%). A higher proportion of households in ELICIT reported owning assets such as benches, mattresses, and mobile phones; having electricity; and having a separate kitchen. The mean enrollment WAZ and HCZ in ELICIT were significantly lower than those on the MAL-ED population (−0.46 difference, 95% CI: −0.59, −0.33 and −0.19 difference, 95% CI: −0.33, −0.05, respectively). On the other hand, the mean LAZ enrollment for ELICIT was significantly higher than that in MAL-ED (0.21 difference, 95% CI: 0.07, 0.35). In comparison to the DHS from the Manyara Region, there was a decrease in improved access to drinking water, sanitation, and assets compared with the households in the Haydom catchment area.

**Table 1:** Baseline characteristics in the ELICIT trial, in comparison to other studies in the same geographical region, during different time periods

	ELICIT	MAL-ED	Demographic and Health Survey
	(2017–2018)	(2009–2014)	(2015–2016)
	(*n* = 1,170)*	(*n* = 250)†	(*n* = 426)
Sociodemographics			
Female gender	573 (49.0)	127 (50.8)	NA
Affiliation with Iraqw tribe	940 (80.3)	226 (90.4)	NA
Firstborn child	205 (17.5)	24 (9.6)	NA
Hospital birth	609 (52.1)	125 (50.0)	NA
Maternal age	27.6 ± 6.6	29.9 ± 6.7	NA
Mother with ≥ 7 years of education	879 (75.1)	149 (59.6)	143/267 (53.6)
Monthly income (/1,000 Tanzanian shillings)	48.8 ± 51.3	43.1 ± 56.2	NA
Risk factors			
Exclusive breastfeeding in the first month	1,105 (94.4)	213 (85.2)	NA
Access to an improved drinking water source	778 (66.5)	102 (40.8)	355 (83.3)
Routine treatment of drinking water	9 (0.8)	15 (6.0)	81 (19.0)
Drinking water > 10 minutes from home	950 (81.2)	243 (97.2)	386 (90.6)
Access to an improved latrine	126 (10.8)	40 (16)	178 (41.8)
Crowding	373 (31.9)	130 (52.0)	NA
Agricultural land ownership	1,128 (96.4)	243 (97.2)	276 (64.8)
Chicken ownership‡	984 (84.1)	157 (62.8)	230 (54.0)
Cow ownership§	793 (67.8)	220 (88)	53 (12.4)
Household assets			
Electricity	398 (34.0)	2 (0.8)	NA
Mattress	667 (57.0)	103 (41.2)	NA
Table	483 (41.3)	73 (29.2)	NA
Bench	988 (84.4)	108 (43.2)	NA
Separate kitchen	725 (62.0)	100 (40.0)	NA
Refrigerator	14 (1.2)	0 (0)	9 (2.1)
Television	76 (6.5)	2 (0.8)	40 (9.4)
Mobile phone	976 (83.4)	149 (59.6)	323 (75.8)
Family bank account	69 (5.9)	11 (4.4)	134 (31.5)
Anthropometry			
Enrollment weight-for-age *z*-score^‖^	−0.60 ± 0.98	−0.14 ± 0.90	NA
Enrollment height-for-age *z*-score¶	−0.79 ± 1.04	−1.01 ± 1.13	NA
Enrollment head-circumference-for-age *z*-score¶	−0.03 ± 1.02	0.16 ± 1.07	NA
Water and sanitation, Assets, Maternal education, and household Income index score (median, interquartile range)^3^	0.30, 0.17	0.23, 0.18	NA

ELICIT = Early Life Interventions for Childhood Growth and Development in Tanzania; MAL-ED = Malnutrition and the Consequences for Child Health; NA = not available. Mean ± SD is shown for continuous variables and the number (percentage) for dichotomous variables unless otherwise stated.

* Includes all enrolled children who completed the first monthly follow-up visit.

† Includes all enrolled children who completed the first demographic questionnaire.

‡ Early Life Interventions for Childhood Growth and Development in Tanzania questionnaires ask only about chicken ownership, and the MAL-ED questionnaires ask whether the household owns chickens and/or ducks.

§ Early Life Interventions for Childhood Growth and Development in Tanzania questionnaire asked if the household owned cows and/or goats.

‖ *N* = 1,164.

¶ *N* = 1,169.

**Figure 2.**
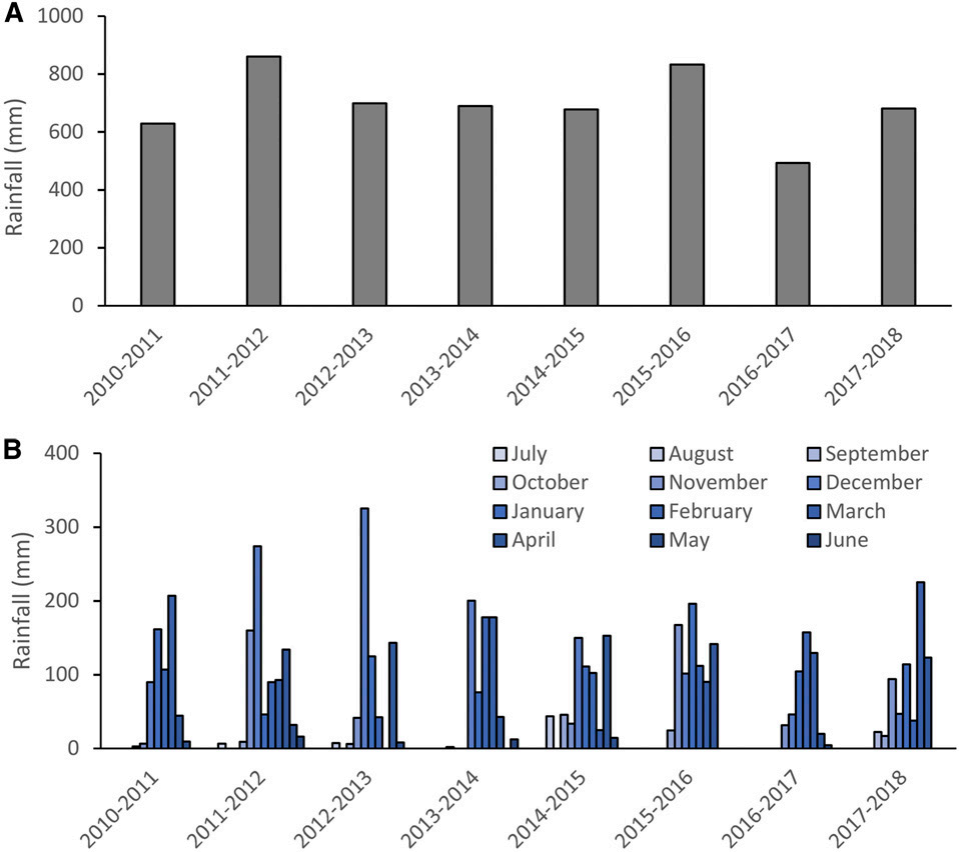
Total seasonal rainfall (**A**) and rainfall by calendar month and year (**B**) in Haydom, Tanzania. This figure appears in color at www.ajtmh.org.

**Figure 3.**
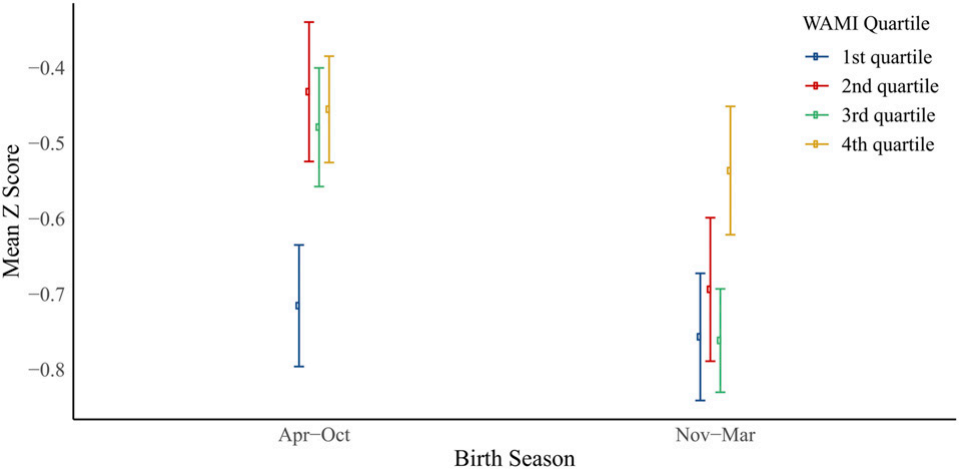
Association between birth season, socioeconomic status as measured by the Water/sanitation, Assets, Maternal education, and Income (WAMI) score, and enrollment weight-for-age *z*-score. This figure appears in color at www.ajtmh.org.

Total rainfall in the year before the ELICIT enrollment period (2017–2018) was particularly low and short (Figure 2), leading to a poorer harvest. We estimated the impact of birth season and other risk factors for enrollment WAZ as a baseline marker or prenatal risk for poor long-term growth outcomes. There was striking seasonality in enrollment WAZ (Table 2), with a nadir in December (−0.64 difference in WAZ from July; 95% CI: −0.89, −0.38). In univariable analysis, birth during the preharvest season was associated with a lower enrollment WAZ (difference in enrollment WAZ for preharvest vs. postharvest: −0.22; 95% CI: −0.33, −0.11) although birth during the pre-harvest season had no association with enrollment LAZ (0.04; 95% CI: −0.08, 0.16). Increasing maternal age (difference per year: 0.02, 95% CI: 0.01, 0.03) and highest quartile WAMI socioeconomic status score compared with lowest quartile (0.24, 95% CI: 0.09, 0.40) were associated with higher WAZ scores. On the other hand, being firstborn was associated with a lower WAZ (−0.55, 95% CI: −0.59, −0.40). There was no association between household food insecurity and enrollment WAZ (−0.02, 95% CI: −0.17, 0.12). Adjusting for birth month, maternal age, WAMI score, and birth order, increasing maternal age was no longer a significant protective factor for enrollment WAZ, although the association with the other factors was not significantly changed (Table 2). Being firstborn and lower maternal age were associated with lower enrollment LAZ. The relationship between birth season and enrollment WAZ varied by WAMI (Figure 3), such that the lowest WAMI score quartile had low WAZ irrespective of birth season, whereas the upper quartiles showed more variation by birth season.

**Table 2:** Sociodemographic factors associated with enrollment WAZ

	Difference in enrollment WAZ (95% CI)		Difference in length-for-age *z*-score (95% CI)	
	Univariable	Multivariable	Univariable	Multivariable
Calendar month				
January	−0.33 (−0.58, −0.08)	−0.37 (−0.61, −0.12)	0.11 (−0.15, 0.38)	0.11 (−0.15, 0.37)
February	−0.32 (−0.58, −0.06)	−0.36 (−0.61, −0.10)	0.26 (−0.02, 0.53)	0.26 (−0.01, 0.53)
March	−0.34 (−0.60, −0.09)	−0.37 (−0.62, −0.11)	0.01 (−0.27, 0.28)	0.00 (−0.27, 0.27)
April	−0.31 (−0.58, −0.03)	−0.31 (−0.58, −0.04)	−0.13 (−0.42, 0.16)	−0.10 (−0.39, 0.19)
May	−0.16 (−0.44, 0.13)	−0.13 (−0.4, 0.15)	−0.02 (−0.31, 0.28)	0.04 (−0.26, 0.33)
June	−0.20 (−0.50, 0.11)	−0.22 (−0.52, 0.08)	−0.01 (−0.33, 0.31)	0.00 (−0.32, 0.31)
July	Ref	Ref	Ref	Ref
August	−0.05 (−0.37, 0.27)	−0.05 (−0.36, 0.27)	−0.08 (−0.41, 0.26)	−0.04 (−0.37, 0.30)
September	−0.35 (−0.76, 0.07)	−0.34 (−0.74, 0.06)	0.69 (0.25, 1.13)	0.67 (0.24, 1.10)
October	−0.47 (−0.78, −0.16)	−0.50 (−0.80, −0.20)	0.48 (0.15, 0.81)	0.49 (0.17, 0.82)
November	−0.43 (−0.73, −0.13)	−0.45 (−0.75, −0.16)	0.08 (−0.24, 0.39)	0.08 (−0.23, 0.39)
December	−0.64 (−0.90, −0.38)	−0.62 (−0.87, −0.37)	0.13 (−0.15, 0.40)	0.15 (−0.12, 0.42)
Maternal age (years)*	0.11 (0.07, 0.15)	0.03 (−0.01, 0.08)	0.13 (0.08, 0.17)	0.09 (0.04, 0.14)
Female gender	−0.06 (−0.17, 0.06)	–	0.06 (−0.06, 0.17)	–
Firstborn	−0.55 (−0.69, −0.40)	−0.54 (−0.71, −0.37)	−0.39 (−0.55, −0.23)	−0.24 (−0.42, −0.06)
Born in hospital	0.10 (−0.02, 0.21)	–	0.10 (−0.02, 0.22)	–
Monthly < 50,000 Tanzanian shillings	−0.09 (−0.22, 0.05)	–	0.06 (−0.08, 0.21)	–
Maternal education ≥ 7 years	0.09 (−0.04, 0.22)	–	0.02 (−0.12, 0.16)	–
Improved sanitation	0.04 (−0.01, 0.08)	–	−0.03 (−0.08, 0.02)	–
Water/sanitation, Assets, Maternal education, and Income score				
Fourth quartile	Ref	Ref	Ref	Ref
Third quartile	−0.11 (−0.26, 0.04)	−0.09 (−0.24, 0.06)	0.00 (−0.16, 0.16)	0.00 (−0.16, 0.16)
Second quartile	−0.08 (−0.25, 0.09)	−0.08 (−0.25, 0.08)	−0.15 (−0.33, 0.03)	−0.15 (−0.33, 0.03)
First quartile	−0.24 (−0.39, −0.08)	−0.28 (−0.43, −0.12)	−0.13 (−0.30, 0.03)	−0.17 (−0.33, 0.00)
Food insecurity	−0.03 (−0.17, 0.12)	–	0.13 (−0.02, 0.28)	–

WAZ = weight-for-age *z*-score.

* Per 5-year increase in maternal age.

Adverse events and SAEs that occurred in the first month of life are shown in Table 3. A child may have had multiple events and each were documented. As expected, the most common AEs were acute lower respiratory infection (ALRI; 38%) followed by upper respiratory tract infection (15%). The most common SAEs were neonatal sepsis (47%) and ALRI (32%). Five neonatal deaths occurred among mothers who had signed consent during pregnancy but before infant enrollment. In univariate analysis, there were no strong risk factors for AEs or SAEs (Table 4). Birth during the preharvest months, lower maternal education, and lower WAMI score all were associated with increased risk of AEs and SAEs, but estimates were imprecise, given low number of events.

**Table 3:** Adverse events in the first month of life in Early Life Interventions for Childhood Growth and Development in Tanzania subjects

Event type	AEs	Serious AEs
	*N* (events per 100 child-months)	*N* (events per 100 child-months)
Any	198 (16.9)	26 (2.2)
Acute lower respiratory tract infection	83 (7.1)	11 (0.9)
Death	5 (0.4)	5 (0.4)
Diarrhea	21 (1.8)	2 (0.2)
Deep vein thrombosis	1 (0.1)	0 (0)
Dysentery	1 (0.1)	0 (0)
Fever	4 (0.3)	0 (0)
Fungal infection	1 (0.1)	0 (0)
Headache	4 (0.3)	0 (0)
Jaundice	1 (0.1)	1 (0.1)
Laboratory abnormality	1 (0.1)	0 (0)
Omphalitis	5 (0.4)	1 (0.1)
Ophthalmological illness	8 (0.7)	0 (0)
Other	22 (1.9)	0 (0)
Partial intestinal obstruction	2 (0.2)	0 (0)
Sepsis	20 (1.7)	16 (1.4)
Skin rash/infection	12 (1)	2 (0.2)
Upper respiratory tract infection	33 (2.8)	1 (0.1)
Urinary tract infection	0 (0)	0 (0)

AEs = adverse events.

**Table 4:** Risk factors for AEs in the first month of life in Early Life Interventions for Childhood Growth and Development in Tanzania

Risk factor	AEs	Serious AEs
	Risk ratio [95% CI]	Risk ratio [95% CI]
During preharvest season (November–March)	1.22 (0.91, 1.66)	1.96 (0.80, 5.49)
Weight-for-age *z*-score at enrollment	0.99 (0.85, 1.15)	0.76 (0.52, 1.17)
Firstborn child	0.97 (0.64, 1.40)	2.35 (0.89, 5.66)
Income < 50,000 Tanzanian shillings	0.97 (0.69, 1.40)	0.91 (0.36, 2.77)
Maternal education ≥ 7 years	0.74 (0.55, 1.03)	0.44 (0.19, 1.08)
Improved sanitation	1.03 (0.92, 1.15)	1.18 (0.86, 1.51)
Water/sanitation, Assets, Maternal education, and Income score		
Third quartile	0.86 (0.59, 1.27)	1.09 (0.36, 3.37)
Second quartile	0.70 (0.43, 1.12)	1.01 (0.26, 3.54)
First quartile	1.10 (0.76, 1.62)	0.75 (0.19, 2.63)
Chicken ownership	0.89 (0.65, 1.24)	0.60 (0.26, 1.53)
Pig ownership	1.14 (0.80, 1.60)	0.92 (0.26, 2.49)
Cow ownership	0.89 (0.67, 1.2)	0.72 (0.28, 1.70)

AEs = adverse events.

## DISCUSSION

We identified several risk factors for enrollment weight in this population used as a marker of prenatal malnutrition. First, in a low-income setting with a unimodal crop cycle, there was a prominent seasonality to birth weight, with lower enrollment weights during the preharvest months. This was more pronounced in households above the first WAMI quartile. These results were also demonstrated in the MAL-ED cohort study where children born during the preharvest months of December to February had a statistically significant decrease in enrollment weight compared with other months.^15^ Maternal weight gain in the second and third trimesters has been previously shown to significantly increase birth weight in a similar setting.^25^ The strong association in our study between the preharvest season and low enrollment weight suggests that maternal nutrition during the third trimester of gestation is particularly critical. Increased diet diversity and nutritional counseling have also been shown to have a positive impact on birth weight.^26,27^ Second, socioeconomic status and birth order were associated with enrollment weight. These factors could modify the impact of the study interventions on child growth and developmental outcomes.

There are potential long-term implications of low birth weight. The prevalence of malnutrition (stunting, wasting, and underweight) is strikingly higher in children with low birth weight,^28^ and these anthropometric measurements correlate with cognitive performance in these and other settings.^29^ This study, in which child growth and development were assessed in a large birth cohort, will provide an opportunity to define the long-term impact of birth season and enrollment weight or both growth and cognitive development. Although the primary outcome of the ELICIT trial is attained length at 18 months, the effect of postnatal malnutrition on length lags behind effects on weight^30^ and thus may be a less sensitive indicator of in utero insult or growth potential at time of birth. Notably, September and October had the highest enrollment lengths, consistent with a 2- to 3-month lag from the highest enrollment weights.

Despite the evidence of sociodemographic improvement in ELICIT compared with the MAL-ED, enrollment WAZ and HCZ were significantly lower. This could be due to the particularly poor harvest that preceded ELICIT enrollment. If seasonal malnutrition was particularly prominent during ELICIT enrollment, this may impact the generalizability of the interventions to other years, even in the same study area. Water, sanitation, and hygiene status also worsened from the MAL-ED to ELICIT, and access to improved WASH was substantially lower in the Haydom area than that in the Manyara region. This, in addition to high exposure to zoonotic pathogens associated with animal husbandry, may increase the force of enteric infections compared with other areas; therefore, the impact of scheduled, interval antibiotic exposure may be weaker than that in areas with a lower force of infection. As noted, the MAL-ED catchment area did not include the more populated Haydom town which may partially account for the secular changes noted between studies.

Interestingly, whereas at the population level, food insecurity was strikingly seasonal (data not shown), at the individual level, there was no association between food insecurity and low enrollment weight. This population-level association was also observed in the MAL-ED cohort.^16^ However, in the MAL-ED, food insecurity was only assessed every 6 months; therefore, individual associations could not be assessed. This suggests that although food insecurity may be a relevant metric of food availability at the population level and, thus, an indicator of seasonal variation in food availability, it may not correlate with food availability at the individual level. For example, worry in this cohort may be a measure of the increased awareness of difficulty accessing food and the resultant motivation to improve it. More specific data on food availability and intake may help further stratify risk factors for low-weight infants.

The AEs and SAEs in the first month occurred before any antibiotic intervention and when mothers had recently begun taking nicotinamide or placebo. As such, these events are likely reflective of the baseline risk in this population. Further analysis of the AEs and SAEs recorded throughout the study period will allow us to assess for any association with the primary interventions and will provide more power to understand the impact of birth season on childhood illness.

Our study has limitations. First, the difference between MAL-ED, ELICIT, and the regional DHS may reflect both socioeconomic improvements and the difference in the study areas, making it difficult to identify the precise reason for any observed differences. Second, defining the preharvest season may vary as this was contingent on food yields from the prior harvest and the timing of the subsequent harvest. We have used a restrictive definition of the preharvest season based on several years of data from the study area, but there may be some misclassification of birth season from year to year. As the derivation of this window is not standardized, it can be difficult to compare across studies. Third, the WAMI score was created from eight diverse sites and may not be the optimal metric for this specific area.^22^ Finally, the categories for AEs were defined by physician review, but broadly, the diagnostic capacity is limited. Furthermore, the power to detect risk factors for early AEs was limited by low event rates.

In summary, the ELICIT cohort was designed to probe potential pathways between early enteric infections, micronutrient availability, and child growth outcomes (LAZ). The study population has substantial variation in baseline characteristics and nutritional status driven by birth season, lower socioeconomic status, and birth order. Further analysis is needed to understand the long-term impacts of seasonal malnutrition, the potential value of maternal interventions to improve low birth weight, and the interactions between these risk factors and the study interventions.
